# A Multiplex RT-PCR Assay for Detection and Differentiation of Avian-Origin Canine H3N2, Equine-Origin H3N8, Human-Origin H3N2, and H1N1/2009 Canine Influenza Viruses

**DOI:** 10.1371/journal.pone.0170374

**Published:** 2017-01-20

**Authors:** Chenxi Wang, Qian Wang, Junyi Hu, Honglei Sun, Juan Pu, Jinhua Liu, Yipeng Sun

**Affiliations:** Key Laboratory of Animal Epidemiology and Zoonosis, Ministry of Agriculture, College of Veterinary Medicine, China Agricultural University, Beijing, China; National Institute for Viral Disease Control and Prevention, CHINA

## Abstract

Virological and serological surveys have documented that H1N1/2009, avian-origin canine H3N2 (cH3N2), seasonal human-origin H3N2 (hH3N2), and equine-origin H3N8 influenza viruses are consistently circulating in dogs. In the present study, a multiplex reverse-transcriptase polymerase chain reaction (mRT-PCR) assay was developed for simultaneous detection and differentiation of these influenza viruses. Four primer sets were designed to target the hemagglutinin genes of H1N1/2009, cH3N2, hH3N2, and H3N8 canine influenza viruses (CIVs). This mRT-PCR assay demonstrated high specificity and sensitivity for the four CIV subtypes. Additionally, mRT-PCR results obtained from 420 clinical samples were consistent with those obtained by the conventional virus isolation method. Our mRT-PCR assay is reliable for clinical diagnosis and rapid identification of CIVs.

## Introduction

Canine influenza virus (CIV) is an emerging pathogen that causes acute respiratory infection in dogs [1,2]. Influenza viruses that originated from avian, equine, and human hosts have transmitted to dogs. Among these viruses, H1N1/2009, avian-origin canine H3N2 (cH3N2), seasonal human-origin H3N2 (hH3N2), and equine-origin H3N8 have resulted in widespread outbreaks [3–6]. H3N8 CIV was first isolated from racing greyhounds that showed respiratory signs in Florida in January 2004 [4]. Phylogenetic analysis suggested that H3N8 CIV was directly transmitted from horses [4], especially considering that H3N8 CIV had spread among several greyhound racetracks in different states and became the dominant CIV subtype circulated in pet dogs in North America [7,8]. The equine H3N8 influenza virus also caused infection in English foxhounds from the United Kingdom in 2002, which emerged independently of the cases from the United States [9]. Since 2007, an avian-origin H3N2 CIV has been detected in domestic dogs in South Korea and China. Experimental infection of cH3N2 CIVs can lead to direct transmission between dogs [2,5,10]. Serological and virological data have also documented the sporadic transmission and subclinical infection of dogs with human influenza H3N2 viruses [3, 11–13]. During the H1N1/2009 pandemic in human, evidence of H1N1/2009 influenza virus infection was found in dogs in Italy and China [6,14]. All eight genes of the two H1N1/2009 viruses isolated from dogs in China were found to be closely related to H1N1/2009 influenza virus circulated in humans, with nucleotide identities of 98.9%–100% to a human representative H1N1/2009 strain, A/California/04/2009 [6]. Results of a recent serological survey showed seropositivity rates of cH3N2, H1N1/2009, and hH3N2 to be 3.5%, 1.5%, and 1.2%, respectively, which emphasized the co-circulation of different CIVs in China [15].

Different CIV subtypes cause highly similar symptoms. Most infected dogs showed clinical signs such as low-grade fever, nasal discharge, and cough [8,11,16,17]. Additionally, co-infection of different influenza viruses in dogs might provide the opportunity for the emergence of novel reassortant influenza viruses. These cases highlight the urgent need to develop a rapid assay that allows prompt identification of different CIV subtypes to monitor public health threats that emerge at the animal-human interface.

Reverse-transcriptase polymerase chain reaction (RT-PCR) has been widely used for influenza virus detection [18,19]. The RT-PCR assay is much quicker and less labor-intensive than conventional methods used for laboratory diagnoses, such as virus isolation followed by subtype determination using hemagglutination inhibition (HI) experiments, immunofluorescence staining, and enzyme-linked immunosorbent assays [20–22]. Furthermore, the multiplex RT-PCR (mRT-PCR) assay can simultaneous detect several pathogens in a single sample with multiple primer sets, which is important for differential diagnoses [23,24].

In this study, an mRT-PCR method was developed by designing primers against the hemagglutinin (HA) gene of different CIV subtypes. Results showed that this method can simultaneously detect and differentiate equine-origin H3N8, cH3N2, hH3N2, and H1N1/2009 CIVs, indicating that it is suitable for routine surveillance and diagnosis of these viruses.

## Materials and Methods

### Ethics statements

All animal work was approved by the Beijing Association for Science and Technology (approval ID: SYXK [Beijing] 2007–0023) and conducted in accordance with the Beijing Laboratory Animal Welfare and Ethics guidelines, as issued by the Beijing Administration Committee of Laboratory Animals, and in accordance with the China Agricultural University Institutional Animal Care and Use Committee guidelines (ID: SKLAB-B-2010-003) approved by the Animal Welfare Committee of China Agricultural University. Viral samples taken from humans were not collected specifically for this study, and no human participants, tissue, or human clinical investigation was involved in this study.

### Viruses

Five H1N1/2009 influenza viruses isolated from human (n = 3) or dogs (n = 2), five cH3N2 viruses isolated from dogs, and five hH3N2 viruses were used in this study (Table 1). As controls, avian-origin H9N2 and H5N1 viruses were also tested in this study because of their demonstrated sporadic infection in dogs in other studies [25,26]. Three viruses known to produce similar symptoms in dogs in China, canine distemper virus (CDV), canine parainfluenza virus (CPIV), and canine adenovirus type 2 (CAV-2), were also tested. The viruses were propagated in the allantoic cavities of 9- to 11-day-old embryonated chicken eggs. The 50% tissue culture infective dose (TCID_50_) for each virus was calculated by the method described by Reed and Muench [27].

**Table 1:** Influenza viruses used in this study.

Virus	Subtype	Source	Accession number of HA gene
A/canine/Beijing/364/2009^a^	H3N2	canine	JX101377
A/canine/Beijing/359/2009	H3N2	canine	JX101393
A/canine/Beijing/362/2009	H3N2	canine	JX101385
A/canine/Beijing/305/2009	H3N2	canine	JX101401
A/canine/Beijing/418/2010	H3N2	canine	JX101369
A/Jiangxi/262/2005^a^	H3N2	human	EU876769
A/Beijing/07/2012	H3N2	human	KF471237
A/Beijing /34/2013	H3N2	human	KJ577143
A/Beijing/369/2010	H3N2	human	KP459340
A/Beijing/332/2009	H3N2	human	CY108834
A/canine/Colorado/6723-8/2008^a^	H3N8	equine	CY067574
A/canine/Iowa/13628/2005	H3N8	equine	DQ146419
A/canine/California/70645-4/2006	H3N8	equine	CY067390
A/canine/Florida/43/2004	H3N8	equine	DQ124190
A/canine/Colorado/866907/2010	H3N8	equine	JX235380
A/canine/Beijing/cau2/2009^a^	H1N1	canine	JN540086
A/California/04/2009	H1N1	human	GQ280797
A/canine/Beijing/cau9/2009	H1N1	canine	JN540094
A/Beijing/7/2009	H1N1	human	HQ533864
A/Beijing/132/2010	H1N1	human	KF918703
A/chicken/Jiangsu/TS/2010	H9N2	avian	KC281007
A/chicken/Sheny/0606/2008	H5N1	avian	JQ277225

^a^ These viruses were used as reference strains in development of the mRT-PCR assay and in the sensitivity assay.

### Generation of H3N8 CIVs by reverse genetics

Because equine-origin H3N8 CIVs have not been found to circulate in China, we rescued five equine-origin H3N8 CIVs in this study (Table 1). The HA gene segments of the five H3N8 CIVs (A/canine/Colorado/6723-8/2008, A/canine/Iowa/13628/2005, A/canine/California/70645-4/2006, A/canine/Florida/43/2004, A/canine/Colorado/866907/2010) and the other seven gene segments of A/canine/Colorado/6723-8/2008 (H3N8) CIV were synthesized according to the sequences in the National Center for Biotechnology Information (NCBI) Influenza Virus Database (http://www.ncbi.nlm.nih.gov/genomes/FLU/Database/nph-select.cgi). The CIVs were generated using reverse genetics, as described previously [28], in which the HA gene was obtained from each of the five H3N8 CIVs and the other seven genes were obtained from A/canine/Colorado/6723-8/2008. In brief, the synthesized viral genes were cloned into the dual-promoter plasmid, pHW2000. 293T cells were transfected with 0.5 μg of each of the eight plasmids and 10 μl Lipofectamine 2000 (Invitrogen, Carlsbad, CA, USA) in a total volume of 1 ml of Opti-MEM (Invitrogen). After incubation at 37°C for 6 h, the transfection mixture was removed and 2 ml of Opti-MEM containing 1 mg/ml of TPCK-trypsin was added to the cells. After 72 h, the supernatant was inoculated into 10-day-old specific pathogen-free embryonated chicken eggs to produce stock viruses. Viral RNA was extracted and analyzed by RT-PCR, and each segment was sequenced to confirm the identity of the virus. TCID_50_ of rescued viruses were determined in MDCK cells.

### Clinical samples

A total of 420 nasopharyngeal swabs were randomly collected from dogs with signs of respiratory disease at Veterinary Teaching Hospital of China Agricultural University during 2012–2015 and stored at -80°C. Clinical samples were taken with informed consent from animal owners. All swabs were placed in 1 ml cold phosphate-buffered saline containing 10,000 U/ml each of penicillin and streptomycin and centrifuged at 8,000 rpm for 5 min. All nasopharyngeal swab specimens were used for the mRT-PCR assay. To compare the mRT-PCR results with those obtained from the conventional virus isolation method, all specimens were also inoculated into 9- to 11-day-old embryonated chicken eggs, then incubated at 35°C for 72 h. Hemagglutination assay and HI assays were used to confirm the CIV subtypes, as described previously [29].

### Primer sequences

HA gene nucleotide sequences of H1N1/2009, cH3N2, hH3N2, and equine-origin H3N8 were downloaded from the NCBI Influenza Virus Resource (www.ncbi.nlm.nih.gov/genomes/FLU) and aligned to identify conserved regions using the MEGA6 program (DNAStar, Inc., Madison, WI, USA). Specific primers to the HA genes of these viruses were designed using Primer Premier 5.0 (Table 2). The primers were analyzed using OLIGO 6.0 software to ensure that they could be used.

**Table 2:** Primers Used for the mRT-PCR Assay.

Specific primers	Primer sequences (5′→3′)	PCR products
H3N8-485F^a^	5′-TCTTTAGCCGACTGAATTGGCTAAC-3′	148 bp
H3N8-632R^b^	5′-ATGTACAATTTTGTCTGCTCTT-3′	
hH3N2-818F	5′-CACAGGGAATCTAATTGCTC-3′	303 bp
hH3N2-1120R	5′-TCCACCATTCCCTCCCAAC-3′	
H1N1-679F	5′-TTCAAGCCGGAAATAGCAATAAGAC-3′	407 bp
H1N1-1085R	5′-ACCATCCCTGTCCACCCCCCTTC-3′	
cH3N2-602F	5′-CAAGCACTAATCAAGAACAAAC-3′	544 bp
cH3N2-1145R	5′-TCTGCTGCTTGTCCTGTACCTT-3′	

^a^F, forward primer.

^b^R, reverse primer.

### Viral RNA and DNA extraction and the mRT-PCR assay

Viral RNA was extracted from infectious allantoic fluid and nasopharyngeal swabs samples using Trizol LS reagent (Invitrogen) according to the manufacturer’s instructions. In brief, 300 μl of allantoic fluid or specimens were mixed with 900 μl of Trizol LS reagent and placed on ice for 10 min. Chloroform (200 μl) was added and the mixture was placed on ice for a further 5 min with occasional inversion. The resulting suspension was centrifuged at 4°C for 15 min at 13,000 rpm. The RNA-containing aqueous phase was precipitated with an equal volume of isopropanol. The mixture was placed at -20°C for 15 min and centrifuged at 13,000 rpm for 15 min at 4°C. The supernatant was removed, and the RNA pellet was washed with 1 ml of 75% ethanol and centrifuged at 8,000 rpm for 5 min at 4°C. RNA was removed using nuclease-free water. Reverse-transcription (RT) reactions were performed using a RevertAidTM First Strand cDNA Synthesis Kit (Fermentas, Vilnius, Lithuania) according to the manufacturer’s protocol. Viral DNA was extracted using an Aidlab DNA Extraction Kit (Aidlab, Beijing, China) according to the manufacturer’s instructions. The PCR mixture for each sample consisted of 12.5 μl 2× EasyTaq PCR SuperMix (Transgen Biotech, Beijing, China), 6.5 μl double-distilled water, 0.5 μl each primer (20 μM/μl of each primer), and 2 μl cDNA or DNA template. PCR comprised an initial denaturation step at 94°C for 5 min, followed by 30 cycles of amplification involving denaturation at 94°C for 30 s, annealing at 57°C for 30 s, and extension at 72°C for 35 s, followed by a final extension step at 72°C for 10 min. The amplified products (5 μl) were loaded onto 2% (w/v) agarose gels containing 5% (v/v) nucleic acid stain. Electrophoresis was conducted using 1× TAE buffer and PCR products were visualized under UV transillumination.

### Assessment of the specificity of the mRT-PCR assay

Four representative strains, A/canine/Beijing/cau2/2009 (H1N1), A/Jiangxi/262/05 (H3N2), A/canine/Beijing/364/2009 (H3N2), and A/canine/Colorado/6723-8/2008 (H3N8), were selected as reference strains to evaluate the specificity of the mRT-PCR assay (Table 1). mRT-PCR was performed using these strains either alone or in combination. Avian-origin H9N2 and H5N1 viruses, which caused sporadic infection in dogs, and canine infectious viruses CDV, CPIV, and CAV-2 were used as controls for the specificity assay. Additional negative controls included non-inoculated allantoic fluid. PCR-amplified products of the expected sizes were purified from agarose gels using an AxyPrep DNA Gel Extraction Kit (Axygen Scientific, Inc., CA, USA) according to the manufacturer’s instructions. Additionally, five unique strains of each of the four CIV subtypes were tested individually (Table 1). The presence of a band of predicted size was indicative of specific amplification for each virus and the RT-PCR products of the expected sizes were sequenced in both directions at the Beijing Genomics Institute (China).

### Assessment of the sensitivity of the mRT-PCR assay

The limits of detection were determined by analyzing a 10-fold dilution series of RNA templates extracted from the four representative strains (Table 1). The individual reference viruses were diluted from 1 × 10^5^ to 1 × 10^−1^ TCID_50_/100 μl, and the mixture of the four viruses was diluted from 1 × 10^5^ to 1 × 10^0^ TCID_50_/100 μl. The detection limits of the mRT-PCR assay for the four individual viruses and a mixture of these viruses were determined.

## Results

### Primers design and selection

The available HA gene nucleotide sequences of different CIV subtypes were analyzed to identify conserved regions. H1N1 CIVs were isolated in 2009 in Beijing; cH3N2 CIVs were isolated from 2006 to 2015 in different countries, including China, Korea, and Thailand; the majority of hH3N2 influenza viruses were isolated from 2008 to 2015 in China, with additional isolates from Korea; and equine-origin H3N8 CIVs were isolated from 2003 to 2015 in Australia and the United States. Phylogenetic analysis showed that each of the eight gene segments of H1N1 CIVs shared a close relationship with H1N1/2009 influenza viruses in humans. For this reason, a combination of HA sequences from H1N1 CIVs and H1N1/2009 influenza viruses isolated from 2009 to 2015 in different countries were also included in the primer design. Various primer combinations were evaluated for amplification efficiency, specificity, and ability to distinguish PCR products by size. Finally, four primers sets were identified with high sensitivity and specificity for detection of HA genes from all four CIV subtypes (Table 2).

### Development of the mRT-PCR assay

**Fig 1:**
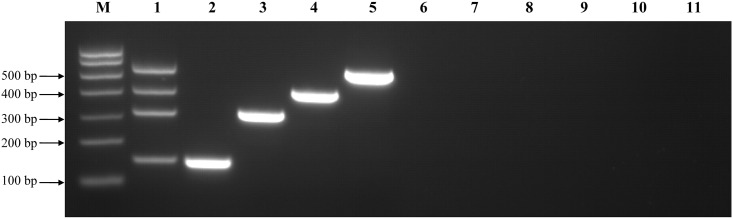
Specificity of the mRT-PCR assay. Lane M: molecular marker. Lane 1: a mixture containing equine-origin H3N8 (A/canine/Colorado/6723-8/2008; 148 bp predicted size), hH3N2 (A/Jiangxi/262/05; 303 bp predicted size), H1N1/2009 CIV (A/canine/Beijing/cau2/2009; 407 bp predicted size), and cH3N2 (A/canine/Beijing/364/2009; 544 bp predicted size) influenza viruses. Lane 2: equine-origin H3N8 CIV (A/canine/Colorado/6723-8/2008). Lane 3: hH3N2 influenza virus (A/Jiangxi/262/2005). Lane 4: H1N1/2009 CIV (A/canine/Beijing/cau2/2009). Lane 5: cH3N2 influenza virus (A/canine/Beijing/364/2009). Lane 6: avian-origin H9N2 influenza virus (A/chicken/Jiangsu/TS/2010). Line 7: avian-origin H5N1 influenza virus (A/chicken/Sheny/0606/2008). Lane 8: CDV (CDV-WZ). Lane 9: CPIV. Lane 10: CAV-2. Lane 11: negative control allantoic fluid.

To establish a multiple RT-PCR assay for the detection of H1N1, cH3N2, hH3N2, and H3N8 CIVs, four primer sets specific to the HA genes of these viruses were used in the mRT-PCR assay (Table 2). The primer sets were designed to amplify (in increasing order of size) a 148-bp fragment from equine-origin H3N8 CIVs, a 303-bp fragment from hH3N2 influenza viruses, a 407-bp fragment from H1N1/2009 influenza viruses, and a 544-bp fragment from cH3N2 CIVs. PCR-amplified products with the same sizes as the reference strains, A/canine/Colorado/6723-8/2008 (H3N8), A/Jiangxi/262/05 (hH3N2), A/canine/Beijing/cau2/2009 (H1N1), and A/canine/Beijing/364/2009 (cH3N2), were visualized on 2.0% (w/v) agarose gels, respectively (Fig 1, Lane 1). The mRT-PCR products were sequenced to assess accuracy (data not shown).

### Specificity of the mRT-PCR assay

**Fig 2:**
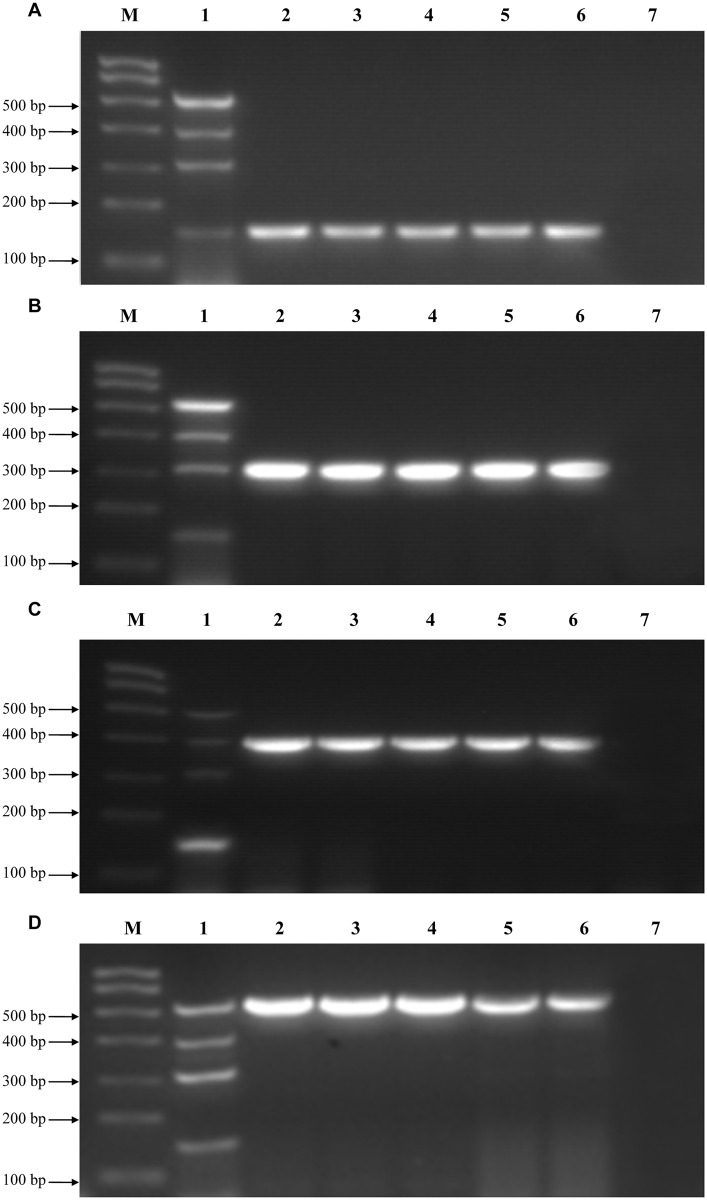
Detection range of the mRT-PCR assay. Detection of the four following strains: (A) equine-origin H3N8 CIVs, (B) hH3N2, (C) H1N1/2009, and (D) cH3N2. Lane M: molecular marker. Lane 1: a mixture containing equine-origin H3N8 (A/canine/Colorado/6723-8/2008; 148 bp predicted size), hH3N2 (A/Jiangxi/262/2005; 303 bp predicted size), H1N1/2009 CIV (A/canine/Beijing/cau2/2009; 407 bp predicted size), and cH3N2 (A/canine/Beijing/364/2009; 544 bp predicted size) influenza viruses. Lane 7: negative control allantoic fluid. (A) Lanes 2–6: A/canine/Colorado/6723-8/2008, A/canine/Iowa/13628/2005, A/canine/California/70645-4/2006, A/canine/Florida/43/2004, A/canine/Colorado/866907/2010; (B) Lanes 2–6: A/Jiangxi/262/2005, A/Beijing/07/2012, A/Beijing/34/2013, A/Beijing/369/2010, A/Beijing/332/2009; (C) Lanes 2–6: A/canine/Beijing/cau2/2009, A/California/04/2009, A/canine/Beijing/cau9/2009, A/Beijing/7/2009, A/Beijing/132/2010; (D) Lanes 2–6: A/canine/Beijing/364/2009, A/canine/Beijing/359/2009, A/canine/Beijing/362/2009, A/canine/Beijing/305/2009, A/canine/Beijing/418/2010.

The specificity of the four primer sets was determined using RNA extracted from the four representative CIV strains. mRT-PCR amplified products of expected sizes were obtained from the mixture of four viral templates and the single viral template using the four primer sets. Each primer pair produced a single amplified band without non-specific amplification (Fig 1). We also tested five unique strains each of H1N1/2009, cH3N2, hH3N2, and H3N8 influenza viruses (Table 1). As expected, all influenza viruses tested were detected at predicted sizes by this assay, and no non-specific amplification was observed (Fig 2A–2D). This suggests that the mRT-PCR assay can detect and differentiate multiple influenza subtypes. The PCR products from each of the 20 viruses were confirmed by sequencing (data not shown). Cross-reactivity was not observed in any of the control viruses (H9N2, H5N1, CDV, CPIV, CAV-2) or negative controls, which indicates that the assay is highly specific to the four CIV subtypes.

### Sensitivity of the mRT-PCR assay

**Fig 3:**
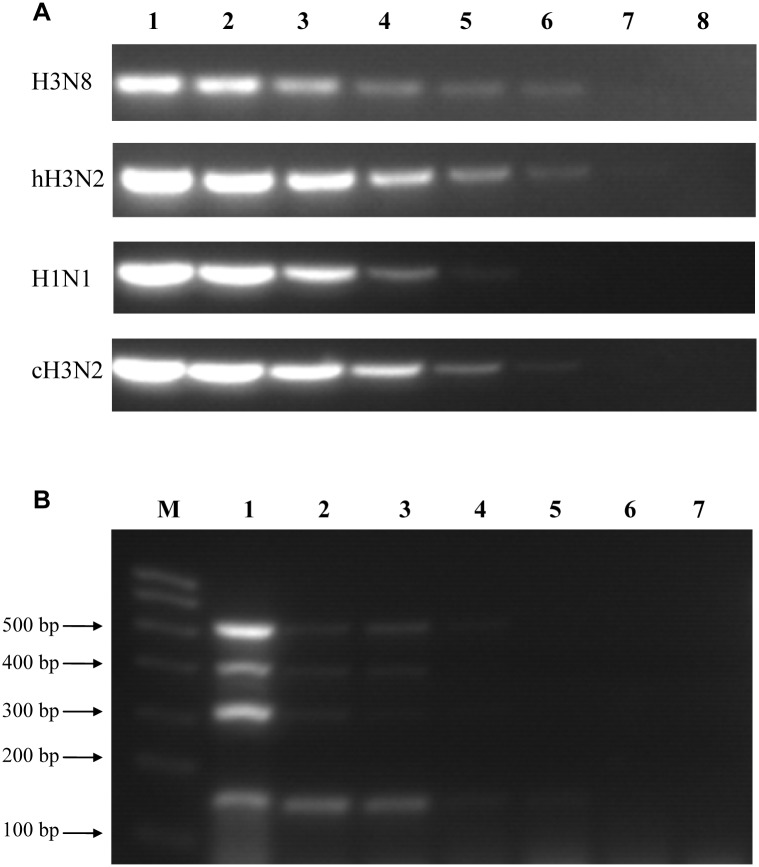
Sensitivity of the mRT-PCR assay. (A) Lanes 1–7: Individual equine-origin H3N8 (A/canine/Colorado/6723-8/2008), hH3N2 (A/Jiangxi/262/2005), H1N1/2009 (A/canine/Beijing/cau2/2009), and cH3N2 (A/canine/Beijing/364/2009) viruses were tested at 10-fold dilutions ranging from 1 × 10^5^ to 1× 10^−1^ TCID_50_/100 μl. Lane 8: negative control allantoic fluid. (B) Lanes 1–6: A mix of the same four viruses were tested using serial 10-fold dilutions from 1 × 10^5^ to 1 × 10^0^ TCID_50_/100 μl. Lane M: molecular marker; Lane 7: negative control allantoic fluid.

The detection limits of the assay were evaluated by using serial 10-fold dilutions of RNA templates extracted from the four representative strains. The lowest concentration detected for H3N8, hH3N2, and cH3N2 viruses was 1 × 10^0^ TCID_50_/100 μl, while the detection limit for H1N1/2009 was 1 × 10^1^ TCID_50_/100 μl (Fig 3A). A mixture of the viruses was also used to determine whether the mRT-PCR assay could be useful for detecting viruses in co-infections. All viruses present in the mixture sample were detectable at a concentration of 1 × 10^3^ TCID_50_/100 μl (Fig 3B). These results suggest that this mRT-PCR assay has high sensitivity.

### Evaluation of the mRT-PCR assay using clinical specimens

To validate the assay with clinical samples, a total of 420 clinical nasopharyngeal swabs obtained from dogs with signs of respiratory disease were tested, and the results were compared to those obtained using conventional methods for laboratory diagnosis including virus isolation in embryonated chicken eggs and hemagglutination/HI assays. Nine of the 420 clinical nasopharyngeal swabs samples were positive for cH3N2 CIVs by mRT-PCR (Table 3). All positive samples identified by the mRT-PCR assay were also confirmed to be positive by traditional virus isolation and identification methods, and no false-positive results were obtained. Results showed a high level of agreement between the mRT-PCR assay and conventional methods. Sequencing results confirmed that these samples corresponded with the expected HA subtypes of the influenza viruses (data not shown).

**Table 3:** Detection of Clinical Specimens by mRT-PCR.

Assay	Target canine influenza viruses			
	H1N1	cH3N2	hH3N2	H3N8
mRT-PCR^a^	0/420^c^	9/420	0/420	0/420
VI^b^	0/420	9/420	0/420	0/420

^a^ mRT-PCR, multiplex RT-PCR.

^b^ VI, virus isolation.

^c^ Data are shown as positive number/total specimens.

## Discussion

Previous studies have showed that multiple viruses of different subtypes and host origins have transmitted to dogs. Particularly, virological and serological surveys have documented that H1N1/2009, cH3N2, hH3N2, and equine-origin H3N8 influenza viruses are consistently circulating in dogs [3–6]. A few methods for detecting equine-origin H3N8 and cH3N2 CIV subtypes have been developed [30–32]. However, no study has described a method for distinguishing different influenza virus subtypes that circulate in dogs. Here, we developed an mRT-PCR assay to simultaneously identify and differentiate H1N1/2009, cH3N2, hH3N2, and equine-origin H3N8 influenza viruses in a single sample that contained almost all of the influenza viruses that circulate in dogs. This method permits one-step detection of the CIVs and enables subtyping of those CIVs when dogs are co-infected with several subtypes.

Traditional virus isolation assays (with HI assay) have some limitations. Firstly, it is time consuming and takes approximately 3–4 days to complete the diagnosis. Secondly, false-negative results may be obtained if antigenic characterization of influenza virus isolates has changed. This mRT-PCR assay requires only 4–5 h to detect CIVs in nasopharyngeal samples with high specificity and sensitivity, which greatly improves efficiency and accuracy. Furthermore, this assay has the additional benefit of using fewer reagents and is relatively cost-effective.

We evaluated the mRT-PCR assay using 420 clinical samples and the results were consistent with those of the conventional virus isolation method, indicating that the assay is accurate and reliable. Interestingly, we detected nine cH3N2 CIVs using the mRT-PCR method. Phylogenetic analysis confirmed that all isolates were of the avian-origin H3N2 CIV lineage and each gene of these viruses shared high homology. The HA genes of the isolates were closely related to A/canine/Liaoning/1585/2010, and the NA genes were closely related to A/canine/Zhejiang/1/2010 (data not shown). Further studies are necessary to determine the biological characteristics of these cH3N2 CIVs. These data also suggest that co-infection of different influenza virus subtypes in dogs is rare during a certain period of time. In our virological and serological survey, no other influenza virus subtypes were identified in Veterinary Teaching Hospital of China Agricultural University [6,15,33]. However, H5N1 and H9N2 viruses caused sporadic infection in dogs in other studies [25,26]. Therefore, it is worth mentioning that, in specific cases, specific primers for M gene should be used before the mRT-PCR method provided in this study to avoid false-negatives for other influenza virus subtypes.

In conclusion, this study developed a simple, rapid, universal, highly sensitive, and highly specific mRT-PCR assay for simultaneous detection of H1N1/2009, cH3N2, hH3N2, and equine-origin H3N8 CIVs. This assay could be used as a reliable tool for clinical diagnosis and surveillance of CIVs worldwide.
